# Case Report: Amelioration of severe metabolic dysfunction-associated steatohepatitis after switching from conventional GLP-1RAs to tirzepatide

**DOI:** 10.3389/fendo.2025.1501984

**Published:** 2025-05-26

**Authors:** Yuki Oe, Takashi Omori, Eriko Aimono, Shin Furukawa, Hirohiko Kitakawa, Masatoshi Tateno, Kiyoshi Sakai, Kyu Yong Cho

**Affiliations:** ^1^ Department of Internal Medicine, Kushiro Red Cross Hospital, Kushiro, Japan; ^2^ Department of Rheumatology, Endocrinology and Nephrology, Faculty of Medicine and Graduate School of Medicine, Hokkaido University, Sapporo, Japan; ^3^ Department of Cancer Pathology, Faculty of Medicine, Hokkaido University, Sapporo, Japan; ^4^ Department of Pathology, Kushiro Red Cross Hospital, Kushiro, Japan; ^5^ Clinical Research and Medical Innovation Center, Institute of Health Science Innovation for Medical Care, Hokkaido University Hospital, Sapporo, Japan

**Keywords:** glucose-dependent insulinotropic polypeptide, glucagon-like peptide 1 receptor, type 2 diabetes, metabolic dysfunction-associated steatohepatitis, obesity

## Abstract

Metabolic dysfunction-associated steatohepatitis (MASH) has cardiometabolic risk factors, such as obesity and type 2 diabetes, and has been reported to have a potentially higher risk of mortality than conventional steatotic liver diseases. Liver fibrosis develops and can progress to cirrhosis and hepatocellular carcinoma. Although some antidiabetic agents have been reported to ameliorate the condition, no specific medical treatment has been developed to date. Tirzepatide is a dual glucose-dependent insulinotropic polypeptide and glucagon-like peptide-1 (GLP-1) receptor agonist (GLP-1RA) that has shown efficacy against MASH in some clinical trials. However, these trials were limited to those with mild-to-moderate fibrosis and their history of treatment was often unclear. Here, we report the case of a 50-year-old man with a 16-year history of diabetes. He demonstrated poor control of his diabetes with elevated liver enzymes. A liver biopsy was performed and he was diagnosed with steatohepatitis. Liraglutide was administered for 3 years but his liver function and glycemic control deteriorated gradually and a second liver biopsy was performed in 2023. The histological examination found cirrhosis and liraglutide was switched to tirzepatide. Over 6 months of administration of tirzepatide, the patient’s glycated hemoglobin and elevated liver enzyme levels improved. A third biopsy was performed, which showed a marked improvement in histology, with the amelioration of liver fibrosis. A diagnosis of steatotic liver disease was made. Although some previous studies had demonstrated an amelioration of liver fibrosis and an improvement in the prognosis of patients following GLP-1RA treatment, effective medications for patients with severe fibrosis or who are refractory to treatment with GLP-1RAs has not been identified to date. We reported a case with severe MASH whose condition had ameliorated by switching from conventional GLP-1RAs to tirzepatide.

## 1 Introduction

Metabolic dysfunction-associated steatotic liver disease (MASLD) is a new concept for steatotic liver disease (SLD), taking cardiometabolic risk factors into account (1). Recent studies have shown that MASLD is associated with higher risks of mortality and metabolic comorbidities than non-alcoholic fatty liver disease (NAFLD) (2). In patients with SLD, the progression of fibrosis can develop severe complications, including hepatocarcinoma (3). Several drugs for the treatment of this challenging disease are in development, but no breakthrough has been made to date (4). Tirzepatide was launched as the world’s first dual glucose-dependent insulinotropic polypeptide (GIP) and glucagon-like peptide-1 (GLP-1) receptor agonist (GLP-1RA) in April 2023 in Japan. The GIP receptor (GIPR) is expressed in various organs, including the brain and adipose tissue, and the infusion of GIPs induces glucose uptake and free fatty acid (FFA) re-esterification secondary to an increase in adipose tissue blood flow ***in vivo***. However, the reported effects of fat mass reduction have been inconsistent and the mechanisms appear to vary over time and according to the nature of any concurrent therapy, especially in combination with GLP-1 RAs (5). In clinical trials, treatment with tirzepatide has been reported to result in an overwhelming reduction in body weight and glycated hemoglobin (HbA1c), implying that it may be beneficial for many obesity-related diseases (6), including MASLD. However, its therapeutic effects for MASLD have not been fully investigated in a clinical setting. Here, we report the case with severe MASLD who had ameliorated by switching from conventional GLP-1RAs to tirzepatide.

## 2 Case description

A 50-year-old man was diagnosed with type 2 diabetes by his primary physician in 2008 and administered medication. Because of a failure of compliance with the medication, diet, and exercise therapy, his HbA1c remained in the 53.0–64.0 mmol/mol range, and he was referred to our department in 2014. At his initial visit, his body weight was 98.9 kg (body mass index: 33.0 kg/m^2^) with an HbA1c of 90.2 mmol/mol with elevated liver enzymes [aspartate aminotransferase (AST), 97 IU/L; alanine aminotransferase (ALT), 109 IU/L]. After referral to our hospital, sodium-glucose cotransporter-2 inhibitor (SGLT2i) was started in 2016 and his HbA1c improved to below 53.0 mmol/mol for some months. However, his glycemic control gradually deteriorated again and the administration of liraglutide was commenced in 2017. Though he had continued liraglutide of 0.9 mg/day for 1 year, it was switched to a dipeptidyl peptidase-4 inhibitor (DPP-4i) for financial reasons. This caused his glycemic control to deteriorate to an HbA1c of 80.3 mmol/mol; therefore, dulaglutide was administered from 2018 as an alternative. During this period of time, elevated liver enzymes persisted. There was no history of alcohol consumption and the results of various antibody tests were negative; therefore, a liver biopsy was performed for further examination in 2020.

**Figure 1:**
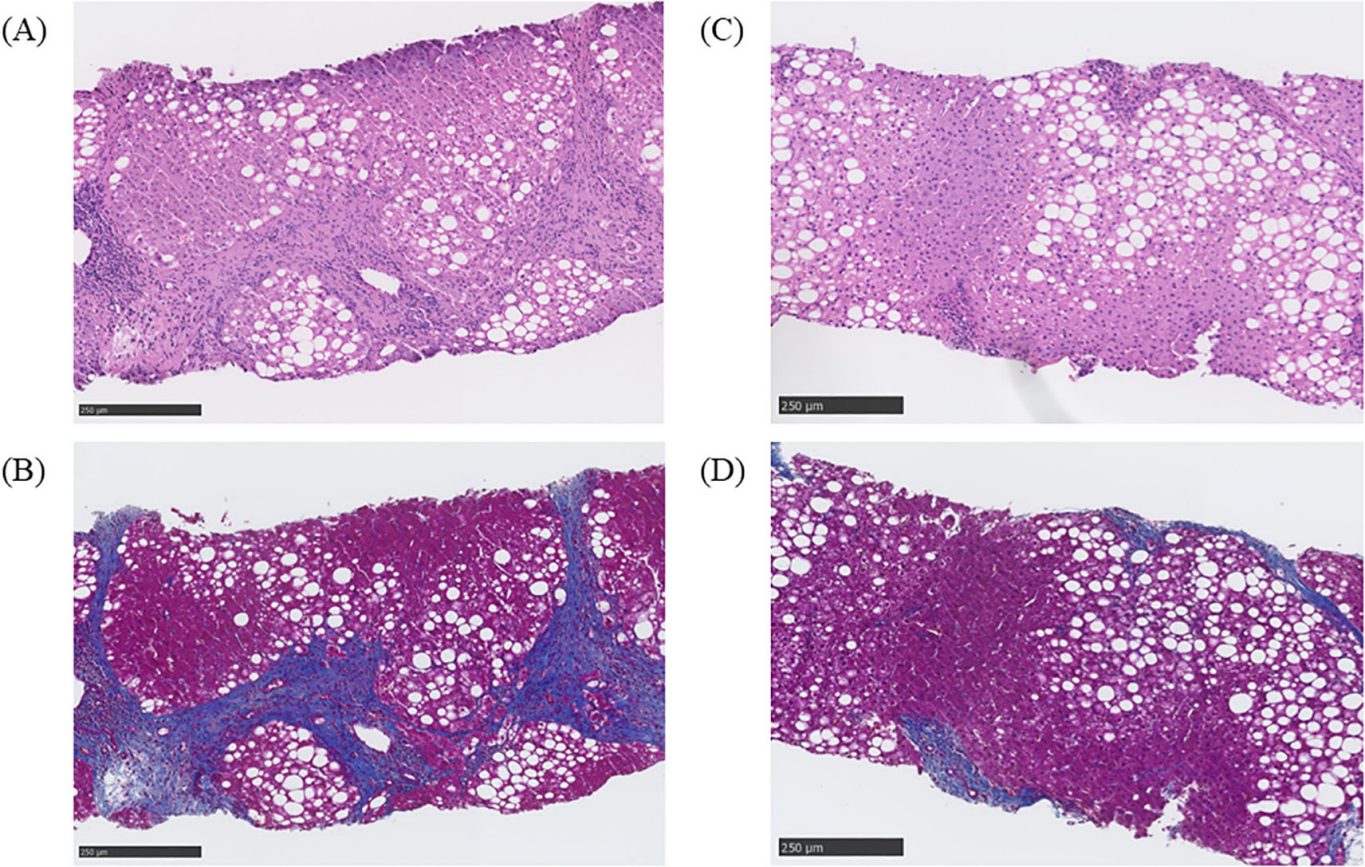
Histological findings before and after treatment with tirzepatide. **(A)** Evaluation of liver steatosis by hematoxylin and eosin staining before administration of tirzepatide. The steatosis was evaluated as follows: non-alcoholic fatty liver disease (NAFLD) activity score (NAS) of 6 (steatosis, 2; lobular inflammation, 2; ballooning, 2). **(B)** Evaluation of liver fibrosis using Masson’s trichrome staining before the administration of tirzepatide.The fibrosis was evaluated as follows: Brunt classification, grade 3, stage 4. **(C)** Evaluation of liver steatosis by hematoxylin and eosin staining after the administration of tirzepatide. The steatosis was evaluated as follows: NAS of 3 (steatosis, 1; lobular inflammation, 1; ballooning, 1). **(D)** Evaluation of liver fibrosis by Masson’s trichrome staining after administration of tirzepatide. The fibrosis was evaluated as follows: Brunt classification, grade 1, stage 2.

**Figure 2:**
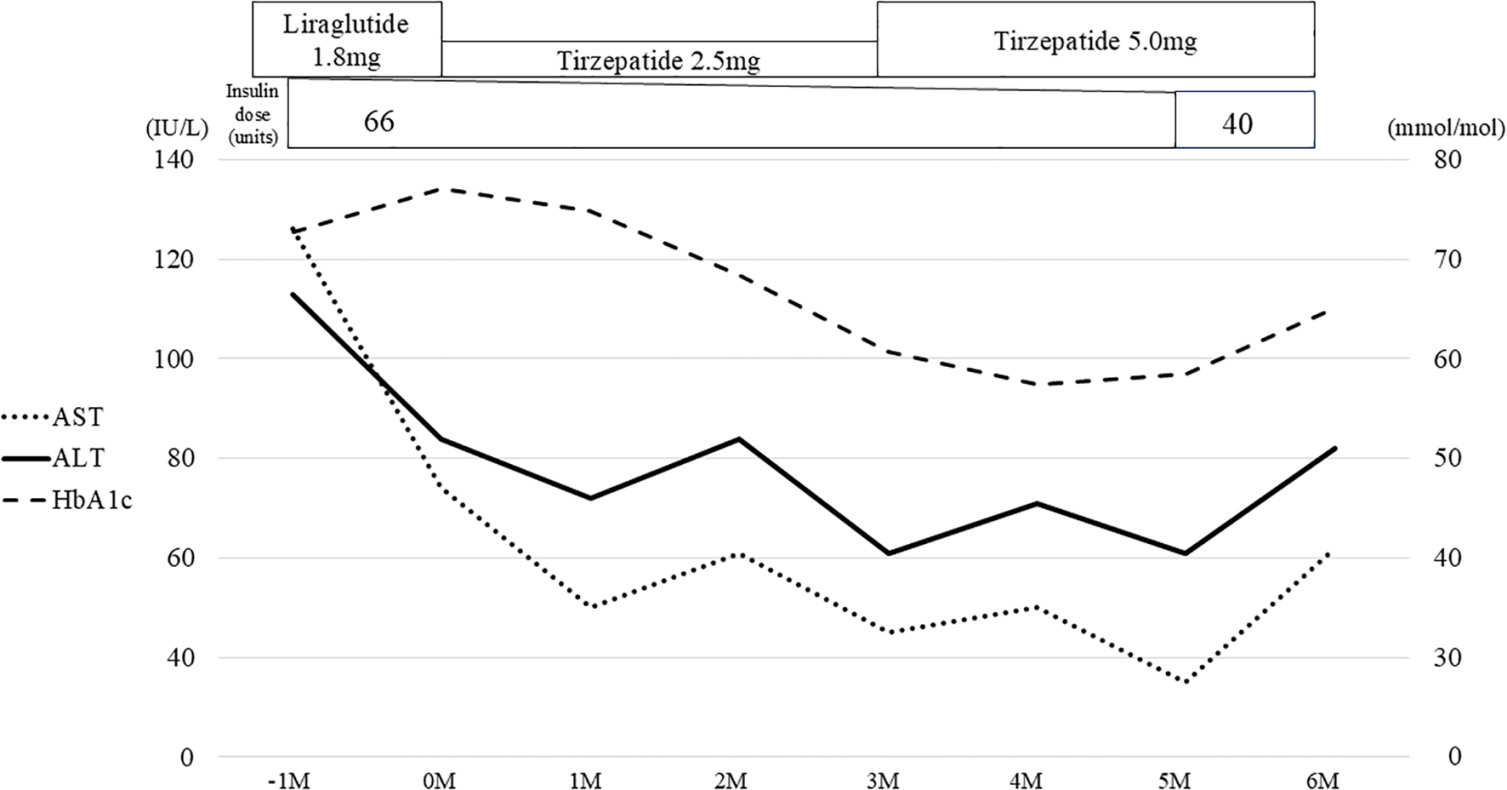
Clinical course associated with tirzepatide for 6 months. The gray bars represent the values of HbA1c, the solid line shows the changes in ALT, and the dotted line shows the changes in AST. Liraglutide was switched to tirzepatide and the concomitant insulin dose was reduced over time. ALT, alanine aminotransferase; AST, aspartate aminotransferase.

The histological diagnosis made was steatohepatitis on the basis of the following evaluations: NAFLD activity score (NAS) of 3 (steatosis, 1; lobular inflammation, 2; ballooning, 0); Matteoni classification, type 2; and Brunt classification, stage 3. In response to this result, the GLP-1RA being administered was switched from dulaglutide to liraglutide 1.8 mg/day, but his HbA1c remained at approximately 70.0 mmol/mol and, finally, insulin therapy was started. Nevertheless, his HbA1c remained over 75.0 mmol/mol and liver enzymes remained high; therefore, a liver biopsy was performed again in 2023. The histological finding had deteriorated, and a diagnosis of cirrhosis was made on the basis of the following evaluations: NAS of 6 (steatosis, 2; lobular inflammation, 2; ballooning, 2), Matteoni classification, type 4; and Brunt classification, stage 4 (**Figures 1A, B**). Following the result, we decided to switch the patient from liraglutide to tirzepatide at 2.5mg weekly. After the initiation of tirzepatide, the patient’s HbA1c improved steadily from 77.1 mmol/mol to 57.4 mmol/mol and he was able to reduce the total daily dose of insulin administered from 66 units to 40 units (**Figure 2**). Furthermore, his elevated liver enzyme levels also decreased (AST from 74 IU/L to 35 IU/L, and ALT from 84 IU/L to 61 IU/L) (**Figure 2**). The dose of tirzepatide being administered was increased to 5.0 mg after 3 months from its initiation (**Figure 2**). Tolerable gastroenterological side-effects were reported during monthly consultations. Although the patient’s body weight temporarily decreased from 92.4 kg to 90.8 kg during the first 3 months of treatment, it returned to 92.0 kg over the following 6 months (**Supplementary Figure 1**). Nevertheless, his visceral fat area decreased from 421.8 cm^2^ to 167.3 cm^2^ over 6 months and his lean mass increased from 59.1 kg to 60.7 kg (**Supplementary Figures 1**, **2**).

After 6 months of treatment, the elevated liver enzyme levels had improved and a third liver biopsy was performed. The histological finding had ameliorated remarkably and a diagnosis of steatotic liver disease was made on the basis of the following evaluations: NAS of 3 (steatosis, 1; lobular inflammation, 1; ballooning, 1), Matteoni classification, type 3; and Brunt classification, stage 2 (**Figures 1C, D**). Some non-invasive tests for liver fibrosis, including fibrosis 4 index (FIB-4 index), NAFLD fibrosis score (NFS), and aspartate aminotransferase-to-platelet ratio index (APRI), also improved (FIB-4 index from 2.99 to 1.66, NFS from 0.093 to -0.07, and APRI from 1.83 to 0.86). As concomitant medications, he had been prescribed antihypertensive drugs, i.e., an angiotensin II receptor blocker and a calcium channel blocker; a proton pump inhibitor; a xanthine oxidase inhibitor; a beta blocker; a histamine-1 receptor antagonist; and ursodeoxycholic acid at constant doses during this period. Diet and exercise therapy had also been continued, with the patient’s energy intake being limited to 1,600 kcal/day (6,694,400 joules per day). The histological finding was evaluated by two professional pathologists in different institutions who were blinded to the laboratory data and there were no discrepancies in their evaluations. The histology was evaluated by representative methods, namely, NAS for steatosis, and Brunt and Matteoni classifications for fibrosis (7–9). Non-invasive tests for liver fibrosis were calculated using the formula presented in the previous report (10).

## 3 Systematic review of the literature

To assess the uniqueness of this case, we conducted a systematic review of the literature in accordance with the Preferred Reporting Items for Systematic Reviews and Meta-Analyses (PRISMA) statement. A systematic review was conducted using the PubMed, Web of Science, and Cochrane databases, searching using the terms “Tirzepatide” and “Liver” on 20 January 2025. We included studies that met the following inclusion criteria, using the Patient, Intervention, Comparison, and Outcome (PICO_ methodology: the targeted patients were all treated with tirzepatide and the intervention was the administration of tirzepatide. We recorded each comparator, but did not establish strict criteria and included case reports. The outcomes were changes in liver function and imaging findings, and adverse events involving the liver were also recorded. We excluded review articles, protocols, practical guidelines, animal studies, expert opinions, abstracts, and commentaries. A PRISMA flow diagram of the selected studies is presented in **Supplementary Figure 3**. A total of 217 publications were identified, from which 77 duplicates were removed. From the remaining 139 articles, 125 were excluded on the basis of a review of their titles and abstracts. From among these articles, studies of hepatic pharmacokinetics and those that did not include the measurement of specific indices of hepatic injury were excluded, even if they were clinical studies.

Following this screening, three case reports, four randomized controlled trials including *post-hoc* analysis, one retrospective observational analysis, and two database analyses remained for inclusion in the systematic review (**Supplementary Table 1**) (11–20). All of the case reports concerned adverse events involving the liver (11–13). The substudy of the SURPASS-3 trial demonstrated an approximate 5.0% reduction in liver fat content on magnetic resonance imaging following the administration of tirzepatide 5.0 mg (15). Hartman et al. reported that biomarkers of liver fibrosis were reduced by tirzepatide (16). Only Loomba et al. reported the resolution of metabolic dysfunction-associated steatohepatitis (MASH) in the SYNERGY-NASH trial, which involved histological investigations (14). However, these subjects were limited to patients with mild-to-moderate fibrosis (Brunt stage 2–3), and patients with cirrhosis were excluded. The only two studies that were not derived from clinical trials were those conducted by Sawamura et al. and Buckley et al. (19, 20). Both showed improvement in liver enzymes, including AST and ALT, but the criteria for determining whether the background status of the liver qualified as MASLD were not clearly defined.

## 4 Discussion

In this case, the switch from liraglutide to tirzepatide demonstrated an improvement in HbA1c with a reduction in the daily insulin dose and showed amelioration of the hepatic steatosis and fibrosis in the histological evaluations. Only a few clinical trials have shown the effect of tirzepatide on SLD to date (15, 16). Significant factors contributing to this change were the reductions in body weight and visceral fat volume (15, 16). However, none of the studies involved histological imaging. Recently, the SYNERGY-NASH trial showed the resolution of MASH by tirzepatide with histological findings (14). However, only a limited number of reports explicitly identified the participants as having MASLD, and very few included biopsy-derived data (14, 16). Thus, we are the first to report the potential utility of tirzepatide for severe MASH with histological evidence and discuss the mechanism involved.

A previous report showed that a body weight reduction of 7%–10% can ameliorate SLD (21). Although the existence of a dose-response relationship between improvements in hepatic steatosis and weight loss has been suggested, there is insufficient evidence that fibrosis is ameliorated by weight loss alone. In other words, the pathogenesis of liver fibrosis comprises various and complex factors from lipid metabolism to chronic inflammation (22, 23). How do the GIP or a combination of GLP-1 and GIP work against these? To date, the expression of GIPR has not been identified in the liver, suggesting that these actions on hepatic metabolism may be indirect (24). Of course, a reduction in energy intake could be expected due to appetite suppression (24, 25). GIPR is widely expressed in the central nervous system, including in the hypothalamus, and one previous study indicated that GIP may act directly by crossing the blood-brain barrier *in vivo* (25). There is a report of the administration of tirzepatide affecting lipid preference, albeit in rodents, which may cause changes not only in the quantity but also in the content of meals (26). The effect of GIP in adipose tissue may be important, but it is controversial. The function of white adipose tissue (WAT) is to regulate circulating lipids through the release or storage of FFAs; however, it can be impaired by various pathways, including decreased adipose tissue blood flow in type 2 diabetes (25, 27). Though an infusion of GIP can cause hypertrophy of adipocytes to occur instead of reducing spillover of FFAs, this may be able to restore the original lipid-buffering capacity by correcting WAT blood flow (28). This optimization of lipid metabolism may have led to a decrease in ectopic fat and improved MASLD. It is also possible that an amelioration of fibrosis was induced *via* the anti-inflammatory action of GIP (24). The administration of GIP reduces the expression of pro-inflammatory cytokines and chemokines, and consequently the inflammation in adipose tissue (29). Inflammatory signals stimulate apoptosis and contribute to the deterioration of liver fibrosis (30). Particularly, GIP may suppress macrophage-driven inflammation (24), and a previous report also showed that tirzepatide inhibits the apoptosis of hepatocytes (16). Even though the present case had severe fibrosis, this was ameliorated by tirzepatide, despite only a 2% weight loss being achieved (**Supplementary Figure 1**). Focusing on the change in fat mass, however, the patient had actually achieved a reduction of 7.0% (**Supplementary Figure 1**). This was consistent with the desired body weight loss, and visceral fat accumulation had also reduced by 15% (**Supplementary Figure 2**). Instead, lean mass had increased by 2.7%, which could explain there being no change in apparent body weight (**Supplementary Figure 1**). Although measurements of inflammatory markers were not made in the present study, it is possible that fat mass loss relieved the inflammation induced by adipose tissue. In addition to the reduction in insulin dosage with favorable glycemic control, the effect on and change in adipose tissue may have contributed to the amelioration of fibrosis. Xiang et al. reported that GLP-1RAs ameliorate muscle atrophy (31), and GIP may help with this. GIPR and GLP-1 receptors are not expressed in skeletal myocytes, but these hormones may have had favorable effects on muscle indirectly (32). Nevertheless, the mechanisms are still uncertain, and therefore, additional research regarding these mechanisms and the durability of the effects of agonism at each receptor, separately and in combination, is warranted.

Of the established antidiabetic agents, pioglitazone, SGLT2is, and GLP-1RAs have been reported to be effective for liver fibrosis (33). The SYNERGY-NASH trial demonstrated the effects of tirzepatide on liver fibrosis, but its effects had not been adequately investigated to date with reference to the backgrounds of the patients. Nearly half of the subjects in this study had type 2 diabetes, but their previous treatments were not presented (14). The combination of type 2 diabetes and NAFLD aggravated each pathogenesis and information regarding the previous treatment is important to assess their potential refractoriness to specific treatments (34). In our case, empagliflozin and liraglutide had previously been administered, but his fibrosis was only ameliorated after the switch from liraglutide to tirzepatide. While the effect on SLD have been reported in SGLT2is and GLP-1RAs, a few studies have involved investigations of their effects on fibrosis using histological assessments (35, 36). Our case also had taken an SGLT2i for a long period of time, and there was no change before and after the administration of tirzepatide. With respect to GLP-1RAs, only liraglutide has been shown to ameliorate liver fibrosis at any stage; semaglutide showed no significant effectiveness in a placebo-controlled trial (36). In compensated liver cirrhosis, the administration of GLP-1RAs has been shown to improve the prognosis of patients, and tirzepatide may have benefits as well (37). In the scope of our systematic review, several studies showed that tirzepatide reduces the level of liver enzymes, even when GLP-1RAs have been used as a pre-treatment. However, the background status of the liver was not described in these reports (**Supplementary Table 1**) (19, 20). A clinical trial (NCT05751720) for severe fibrosis is ongoing, and we hope that this case will be replicated in this trial.

As for limitations, this was a case report and additional clinical trials are desirable in a clinical setting. Liver biopsies were performed using only one puncture at each time point, and there was a risk of sampling errors. We did not examine several biomarkers for the assessment of fibrosis, such as type IV collagen 7S or mac-2-binding protein glycosylation isomer. Quantitative image findings, including elastography or Fibroscan, were not performed either, as the facility did not have the equipment. However, histopathological findings are the most robust means of assessing the status of the liver. Some non-invasive tests, while considered supplementary, were included in the evaluation. Additionally, it should also be noted that there are no current guidelines recommending the use of tirzepatide for the treatment of cirrhosis. We also conducted a systematic review following the PRISMA guidelines, but we did not register it in the database. Though information was obtained from multiple databases, the number of reports extracted was small. These also included case reports, making it unsuitable for standardizing the quality of the studies. Furthermore, the screening was performed by several independent authors without the use of automated software, which may introduce a potential risk of bias. As a potential confounding factor, we could not entirely exclude the possibility of lifestyle changes accompanying medication adjustments, and the retrospective nature of the study made it challenging to minimize sources of bias. Although it is inexplicable that there was no history of pioglitazone administration in the present case, we have shown the efficacy of tirzepatide following treatment with SGLT2is and GLP-1RAs. From the report of Rosenstock et al., patients who exhibit greater improvements in HbA1c may also have more significant improvements in liver impairment (18). Tirzepatide may ameliorate MASH through its marked effects on glycemic control and weight loss, which occur regardless of the previous treatments.

In conclusion, the present case report shows that tirzepatide may be an effective treatment for cases with severe hepatic fibrosis that are refractory to conventional treatment with GLP-1RAs. The combination of type 2 diabetes and liver cirrhosis has a poor prognosis; thus, medications are desired to be effective for both conditions simultaneously. We hope that new solutions will be discovered for this challenging disease.

## Data Availability

The original contributions presented in the study are included in the article/**Supplementary Material**. Further inquiries can be directed to the corresponding author.

## Supplementary material

Supplementary Figure 1 Changes in body composition over 6 months. These data were obtained using a body composition analyzer (InBody 770; InBody USA, Cerritos, CA, USA). The bars show the mass of each tissue compartment; the dotted area, fat mass; the striped area, the lean mass; and the filled black area, other compartments. The mass of each compartment and the total mass are shown for each time point.

Supplementary Figure 2 Changes in each fat mass over 6 months. These data were measured by computed tomography. Within the pie chart, the dotted slice represents the subcutaneous fat mass and the striped area represents the visceral fat mass. The total mass (presented above) reduced over 6 months and, particularly, visceral fat mass predominantly decreased.

Supplementary Figure 3 Preferred Reporting Items for Systematic Reviews and Meta-Analyses (PRISMA) flow diagram. YO and KYC contributed to the screening and selection process of articles manually. Case reports were included.
