# Supplementary figures and images for: Case Report: Amelioration of severe metabolic dysfunction-associated steatohepatitis after switching from conventional GLP-1RAs to tirzepatide
Source: Front Endocrinol (Lausanne). 2025 May 26;16:1501984. doi: 10.3389/fendo.2025.1501984 (PMC12146166; doi:10.3389/fendo.2025.1501984)

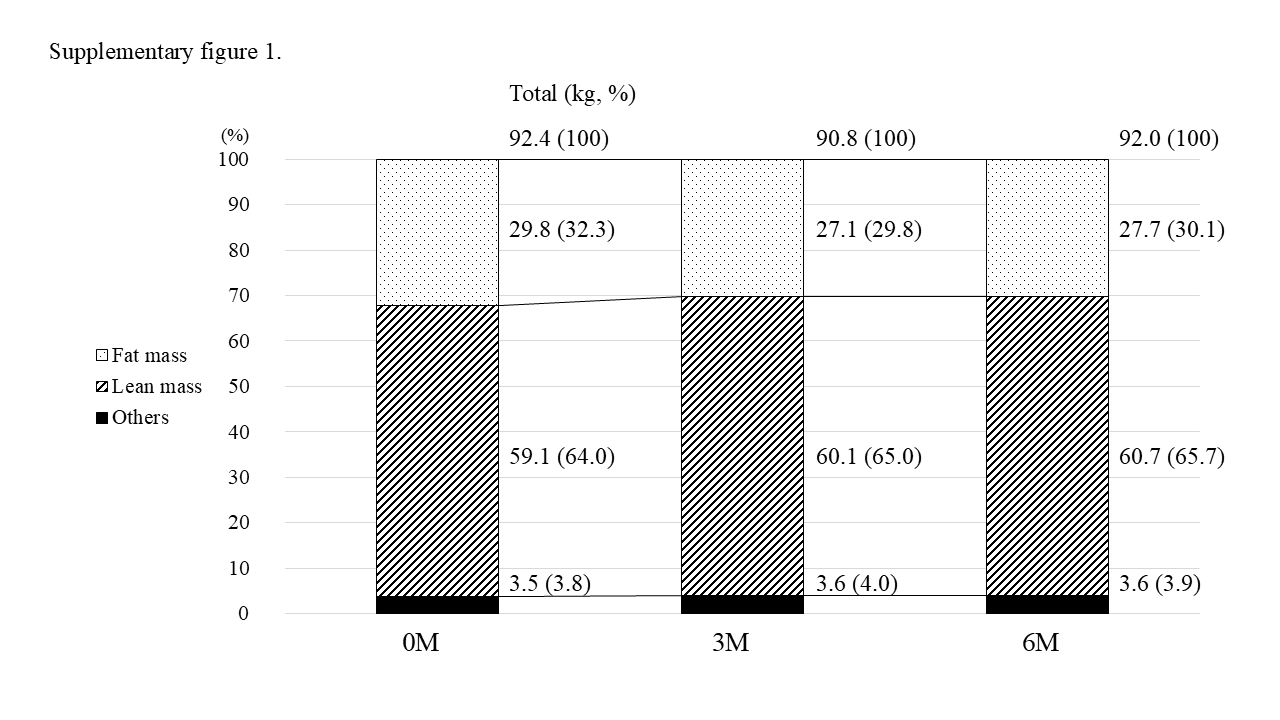

Supplement: Supplementary Figure 1 — Changes in body composition over 6 months. These data were obtained using a body composition analyzer (InBody 770; InBody USA, Cerritos, CA, USA). The bars show the mass of each tissue compartment; the dotted area, fat mass; the striped area, the lean mass; and the filled black area, other compartments. The mass of each compartment and the total mass are shown for each time point. [file Image1.tif]

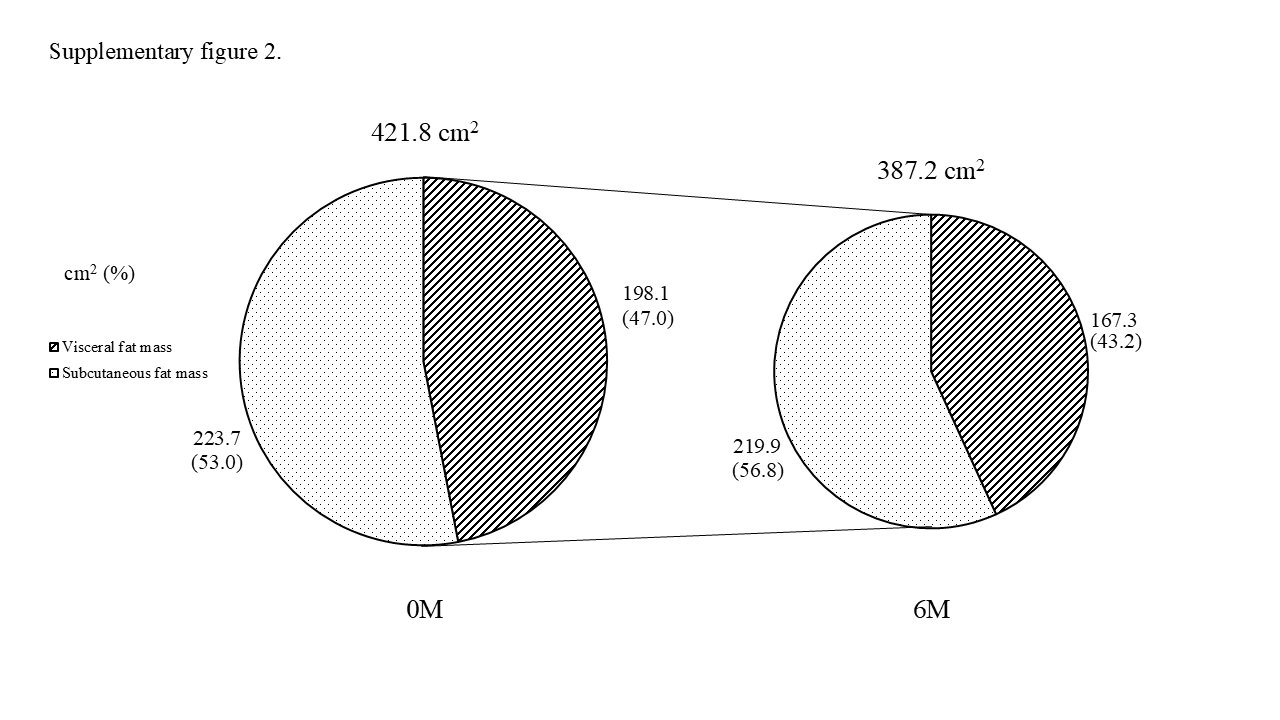

Supplement: Supplementary Figure 2 — Changes in each fat mass over 6 months. These data were measured by computed tomography. Within the pie chart, the dotted slice represents the subcutaneous fat mass and the striped area represents the visceral fat mass. The total mass (presented above) reduced over 6 months and, particularly, visceral fat mass predominantly decreased. [file Image2.tif]

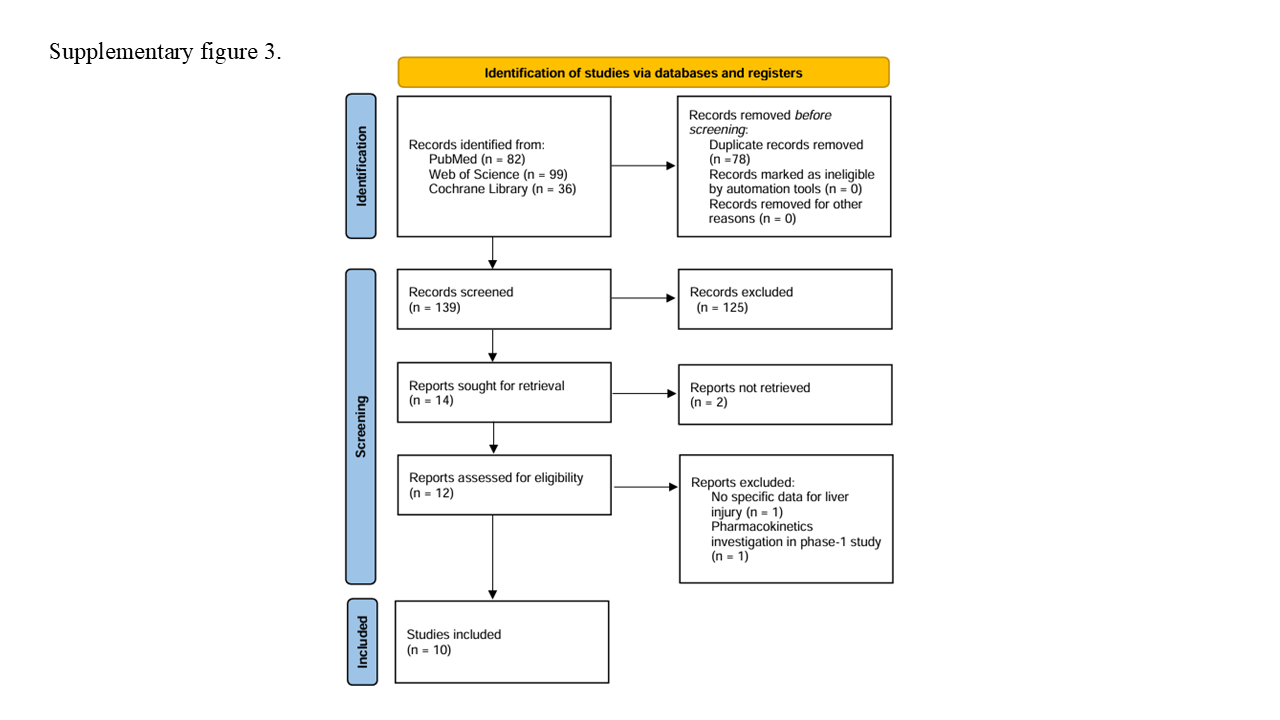

Supplement: Supplementary Figure 3 — Preferred Reporting Items for Systematic Reviews and Meta-Analyses (PRISMA) flow diagram. YO and KYC contributed to the screening and selection process of articles manually. Case reports were included. [file Image3.tif]
