# Supplementary material for: Case Report: Amelioration of severe metabolic dysfunction-associated steatohepatitis after switching from conventional GLP-1RAs to tirzepatide
Source: Front Endocrinol (Lausanne). 2025 May 26;16:1501984. doi: 10.3389/fendo.2025.1501984 (PMC12146166; doi:10.3389/fendo.2025.1501984)
Supplement: Supplementary file 4 [file Table1.docx]

Supplementary table 1. Summary of previous reports on liver outcome in tirzepatide.

| Author | Year | Biopsy | Outcomes | Comparators | Results | Background status of MASLD |
| --- | --- | --- | --- | --- | --- | --- |
| Sawamura T, et al | 2024 | No | AST, ALT, and γGTP levels; FIB-4 index  (Secondary) | Dulaglutide as pre-treatment | The AST, ALT, and γGTP levels significantly decreased after 6 months of tirzepatide treatment. However, the FIB-4 index remained unchanged. | Unspecified. |
| Sohal A, et al. | 2024 | Yes | ALT and AST | None | Induced hepatotoxicity. | Unspecified. |
| Abdullah I, et al. | 2024 | Yes | ALT and AST | None | Induced hepatotoxicity. | Unspecified. |
| Klein JA, et al. | 2024 | No | ALT and AST | None | Possibility of affecting thiopurine metabolism. | Unspecified. |
| Buckley A, et al. | 2024 | No | AST, ALT,  (Secondary) | Unspecified, though 87.8% of subjects were directly switched from GLP-RAs. | Only ALT showed significant reduction. | Unspecified. |
| Loomba R, et al. | 2024 | Yes | The resolution of MASH without worsening of fibrosis (defined as no increase in the fibrosis stage) at week 52. | Placebo | The resolution of MASH without worsening of fibrosis was 44% in the 5-mg tirzepatide  group, 56% in the 10-mg tirzepatide group, and 62% in the 15-mg tirzepatide group. | Histological stage 2 or 3 fibrosis, based on a histological scoring system for nonalcoholic fatty liver disease, including Brunt classification. |
| Cariou B, et al | 2024 | No | Standardized normal z-scores of liver fat measured by MRI. | Insulin glargine | Tirzepatide-treated participants had significant decreases in z-scores of LF (−0.54 [0.84]; p < 0.001). | This was a sub-study of report by Gastaldelli A et al. |
| Rosenstock J, et al. | 2023 | No | AST, ALT,  (Secondary) | Tirzepatide  (Investigation based on HbA1c in pooled data) | The HbA1c <5.7% group had a mean ALT change of −25.4% vs. −15.0% in the 5.7–6.5% group or −0.7% in the >6.5% group at week 40.  The HbA1c <5.7% group had a mean AST change of −10.6% vs. −4.3% in the 5.7–6.5% group or 4.7% in the >6.5% group at week 40. | Unspecified. |
| Gastaldelli A et al | 2022 | No | The change from baseline in LFC measured by MRI-PDFF. | Insulin glargine | The absolute reduction in LFC at week 52 was significantly greater for the pooled tirzepatide 10 mg and 15 mg groups (-8·09%, SE 0·57) versus the insulin degludec group (-3·38%, 0·83). | Baseline LFC was evaluated; however, the proportions of participants with LFC lower than 6% at baseline ranged from 8% to 20%. |
| Hartman ML, et al | 2020 | Partially | Changes from baseline in ALT, AST, K-18, Pro-C3 | Placebo | Significant reductions from baseline in ALT (all groups), AST (all groups except tirzepatide 10 mg), K-18 (tirzepatide 5, 10, 15 mg), and Pro-C3 (tirzepatide 15 mg) were observed at 26 weeks. | Unspecified. |

ALT, alanine aminotransferase; AST, aspartate aminotransferase; FIB-4index, fibrosis-4 index; K-18, keratin-18; LF, liver fat; LFC, liver fat content; MASH, metabolic dysfunction–associated steatohepatitis; MASLD, metabolic dysfunction-associated steatotic liver disease; MRI-PDFF, magnetic resonance imaging-proton density fat fraction; Pro-C3, procollagen III;
